# Systemic Toxicity of Intraperitoneal Vancomycin

**DOI:** 10.1155/2016/3968690

**Published:** 2016-10-20

**Authors:** Teerath Kumar, Iris Teo, Brendan B. McCormick

**Affiliations:** ^1^Division of Nephrology, Department of Medicine, University of Ottawa and The Ottawa Hospital, Ottawa, ON, Canada; ^2^Kidney Research Centre, University of Ottawa, Ottawa, ON, Canada; ^3^Department of Pathology, University of Ottawa and The Ottawa Hospital, Ottawa, ON, Canada

## Abstract

Intraperitoneal vancomycin is used for empiric treatment of peritoneal dialysis peritonitis. It is dosed intermittently and a high systemic concentration is often achieved. Despite this, there are very few reports of systemic toxicity from intraperitoneal vancomycin. We report the course of a patient who developed a drug reaction with eosinophilia and systemic symptoms (DRESS) syndrome after three weeks of intraperitoneal vancomycin. We review the literature and conclude that this is the first ever reported case of DRESS syndrome from intraperitoneal vancomycin.

## 1. Background

Intraperitoneal (IP) vancomycin is frequently used for empiric treatment of peritoneal dialysis (PD) peritonitis and courses of 2 to 4 weeks are commonly used to treat infections with methicillin resistant staphylococcal species. Due to the slow clearance of vancomycin in advanced renal failure, intermittent dosing of vancomycin is used and doses are typically administered every 3 to 5 days [1]. We have recently reported that higher than recommended trough levels of vancomycin are associated with lower rates of relapse of coagulase-negative staphylococcus (CNS) peritonitis and recently some practitioners have been aiming for higher trough levels when treating PD peritonitis due to CNS or methicillin resistant staphylococcus aureus (MRSA) [2]. To date, there has been very little concern about the systemic toxicity of IP vancomycin. We report a case of a severe hypersensitivity reaction to IP vancomycin and review the published literature for reports of toxicity from IP vancomycin.

## 2. Case

A 45-year-old male with end stage renal disease due to chronic glomerulonephritis started automated PD in February 2015 and presented 7 months later with nausea and cloudy PD effluent. The peritoneal effluent white cell count was 996 cells/mL, with a polymorphonuclear cell fraction of 56%. The peritoneal fluid was sent for microbiological investigations and he was given IP vancomycin 2 g and ceftazidime 1.5 g as per the hospital empiric protocol. The culture grew coagulase-negative staphylococcus resistant to beta lactams but sensitive to vancomycin. The IP ceftazidime was discontinued after 2 doses and he continued IP vancomycin every 4 days for a total of 3 weeks. The vancomycin dose was adjusted according to serum vancomycin level and the level remained greater than 25 mg/L throughout the treatment with the highest level being 35 mg/L. His levels were kept deliberately high as he was due for a live donor kidney transplant the following month and the treating team wanted to minimize the risk of relapsed peritonitis.

**Figure 1:**
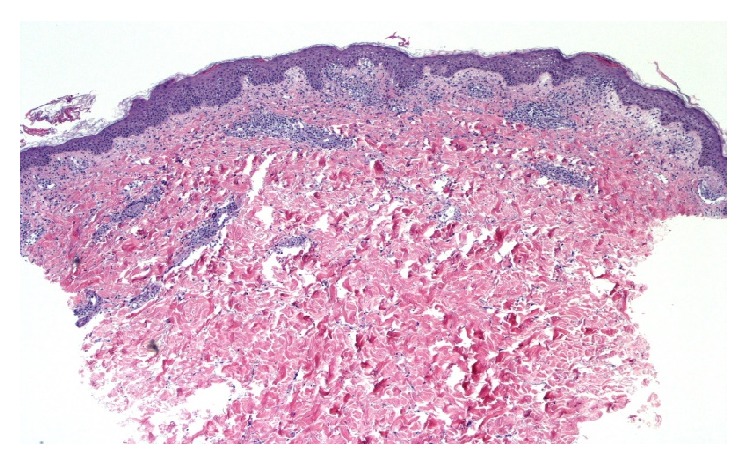
A moderate superficial perivascular infiltration of lymphocytes with some eosinophils and some papillary dermal edema.

**Figure 2:**
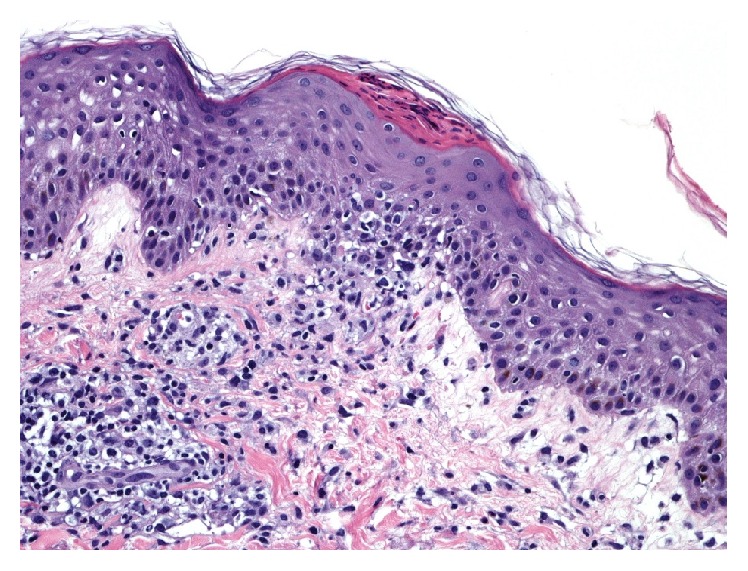
Focal lichenoid inflammation at the dermal-epidermal junction, mild spongiosis, and focal areas of parakeratosis.

One day after completing the course of vancomycin, the patient presented with general malaise and a skin rash which had begun on the abdomen and progressed to involve the entire trunk and proximal limbs. He reported intense pruritus and pain. He was hypotensive with mean arterial blood pressure of 50 mm Hg, heart rate 115, and temperature *T* 39.1°C. There was a diffuse blanchable papular rash involving the trunk and proximal limbs without blistering or sloughing. There was no mucous membrane involvement and no lymphadenopathy. He was admitted to the intensive care unit for hypotension requiring vasopressors and was initially treated for possible sepsis with intravenous saline and broad spectrum antibiotics including vancomycin. Initial laboratory investigations are shown in Table 1. All imaging was normal, and blood and urine cultures revealed no growth. After admission day 2 he had a skin biopsy performed which revealed a moderate mixed perivascular infiltrate, composed mostly of lymphocytes with a few neutrophils and scattered eosinophils (Figure 1). There was a moderate amount of both vacuolar and a slightly lichenoid interface dermatitis, and a few necrotic keratinocytes were seen within the spongiotic epidermis. A few minute foci of parakeratosis were present (Figure 2). PAS stain was negative for fungal organisms. Biopsy findings were compatible with a drug reaction, and clinically he was felt to meet criteria for drug reaction with eosinophilia and systemic symptoms (DRESS syndrome). He was given one dose of intravenous solumedrol 80 mg and then switched to oral prednisone 50 g daily for 5 days. He responded well to corticosteroid therapy and was discharged in stable condition after 3 days and received a live donor kidney transplant 7 weeks later.

## 3. Discussion

Despite over 3 decades of use in the treatment of PD peritonitis, there are very few reports of severe adverse effects from IP vancomycin. We report a case of intraperitoneal vancomycin induced hypersensitivity with severe systemic symptoms including hypotension requiring vasopressor support. We feel it is unlikely that ceftazidime was the culprit drug and subsequently the patient has been challenged with cephalosporins without any reaction. There has only been one previous report of intraperitoneal vancomycin induced hypersensitivity with systemic symptoms, a recent report from South Korea where a patient developed throat tightness and rash [3] (Table 2). In that report, there was no hypotension and the rash was not biopsied.

Intravenous vancomycin can cause several types of adverse reactions, the most common of which is the histamine release syndrome associated with rapid infusions [6]. This phenomenon, known as red man syndrome (RMS), is a type A drug reaction as it can affect any individual given a rapid enough rate of vancomycin infusion. For example, an intravenous infusion rate of vancomycin of 1 g over 10 minutes has been shown to produce RMS in 100% of patients [7]. There is a single report of RMS from intraperitoneal vancomycin in a pediatric patient but no reports in adults [4]. Pediatric patients may absorb vancomycin faster than adults and have a much lower volume of distribution which may be the predisposing factor in the reported case [8]. There are no reports of this phenomenon with IP vancomycin in adult patients, presumably related to the slow rate of absorption of vancomycin through the peritoneal membrane. In adult PD patients, the bioavailability of IP vancomycin following a 4- to 6-hour exchange is between 30 and 70%, a major factor limiting a rapid rise in plasma levels [9].

Type B (hypersensitivity) reactions are also reported with intravenous vancomycin. Immunoglobulin E (IgE) mediated anaphylaxis is well reported and occurs immediately in patients previously exposed to the drug, but among those previously unexposed it can occur after several days of exposure as it takes time for the drug specific IgE antibodies to form [10]. Typical symptoms include urticaria, angioedema, pruritus, tachycardia, hypotension, nausea, vomiting, and hypotension. Our patient certainly had some of these symptoms but in addition had a prominent rash with lymphocytic and eosinophilic infiltrate and we feel his presentation is most consistent with DRESS syndrome. DRESS syndrome is felt to be a separate entity from IgE mediated anaphylaxis and is characterized by T cell activation which is often accompanied by eosinophilia along with high fever and multiorgan involvement as was seen in our patient [11]. DRESS syndrome often presents after weeks of exposure to a medication and responds to corticosteroids in keeping with our patient's course.

With respect to local complications of IP vancomycin, there is a single report of eosinophilic peritonitis due to IP vancomycin (Table 2) [5]. This was diagnosed after a patient developed asymptomatic peritoneal eosinophilia after 7 days of therapy for coagulase-negative staphylococcal peritonitis. There are a number of older reports of chemical peritonitis from IP vancomycin which were attributed to impurities in the vancomycin preparations which were common in an earlier era [12, 13].

We can only speculate whether our patient's high vancomycin levels predisposed him to DRESS syndrome. While the link between intravenous vancomycin exposure and DRESS is well established, none of the large case series report whether high vancomycin levels were present in these cases [14]. This is an issue of particular relevance in peritoneal dialysis as relatively high blood levels of vancomycin are required to ensure that adequate back diffusion of vancomycin to the peritoneal space occurs [2]. PD peritonitis is a localized infection and high IP levels of antibiotics are necessary to achieve adequate clearance of bacteria from the PD catheter. Patients with short dwells, such as those on automated PD, may not achieve adequate IP vancomycin levels while cycling 8–10 hours overnight. Despite this theoretical concern, relapse rates do not appear to be higher among patients treated with automated PD compared with continuous ambulatory PD [15]. An alternative IP vancomycin dosing strategy has been proposed that involves 25 mg/L of vancomycin in each exchange rather than large doses given every few days [9]. This has the advantage of achieving higher intraperitoneal concentrations and preventing potentially risky systemic levels, though it is certainly more onerous. There have not been any studies assessing the efficacy of this approach.

To our knowledge, this is the first report of DRESS syndrome from IP vancomycin. We feel that the lack of awareness of this condition and its possible link to IP vancomycin delayed the diagnosis and resulted in inappropriate empiric therapy with broad spectrum antibiotics including vancomycin. We hope that this case report draws attention to this and other potential complications of IP vancomycin.

## Figures and Tables

**Table 1:** Initial laboratory investigations.

Description	Results	Reference range
WBC	9.8 × 10^9^/L	3.5–10.5 × 10^9^/L
Neutrophil	7.8 × 10^9^/L	2.0–7.5 × 10^9^/L
Eosinophil	0.8 × 10^9^/L	0.0–0.5 × 10^9^/L
Hemoglobin	91 g/L	125–170 g/L
Bilirubin total	5 *μ*mol/L	3–17 *μ*mol/L
AST	19 U/L	15–37 U/L
ALT	22 U/L	17–63 U/L
ALP	39 U/l	50–136 U/L
GGT	32 U/L	15–85 U/L
Lactate	3.3 mmol/L	0.4–2.0 mmol/L

**Table 2:** Reported local and systemic reactions to IP vancomycin.

Case	Patient age (years)	Local or systemic reaction	Time after initiation of IP vancomycin	Diagnosis
Current case	45	Systemic	21 days	DRESS syndrome
Hwang et al. [3]	49	Systemic	7 days	IgE mediated hypersensitivity
Domis and Moritz [4]	11	Systemic	45 minutes	RMS
Rosner and Chhatkuli [5]	61	Local	7 days	Eosinophilic peritonitis

## Additional Points

Intraperitoneal vancomycin is occasionally associated with local and systemic complications. And practitioners need to maintain vigilance with the use of this medication.
